# Chitosan Oleate Salt as an Amphiphilic Polymer for the Surface Modification of Poly-Lactic-Glycolic Acid (PLGA) Nanoparticles. Preliminary Studies of Mucoadhesion and Cell Interaction Properties

**DOI:** 10.3390/md16110447

**Published:** 2018-11-15

**Authors:** Dalila Miele, Silvia Rossi, Giuseppina Sandri, Barbara Vigani, Milena Sorrenti, Paolo Giunchedi, Franca Ferrari, Maria Cristina Bonferoni

**Affiliations:** 1 Department of Drug Sciences, University of Pavia, 27100 Pavia, Italy; dalila.miele01@universitadipavia.it (D.M.); silvia.rossi@unipv.it (S.R.); giuseppina.sandri@unipv.it (G.S.); barbara.vigani@unipv.it (B.V.); milena.sorrenti@unipv.it (M.S.); franca.ferrari@unipv.it (F.F.); 2 Department of Chemistry and Pharmacy, University of Sassari, 07100 Sassari, Italy

**Keywords:** chitosan oleate salt, amphiphilic polymer, PLGA, nanoparticles, mucoadhesion, Caco-2 cell culture, nile red, curcumin

## Abstract

Most of the methods of poly-lactic-glycolic acid (PLGA) preparation involve the passage through the emulsification of a PLGA organic solution in water followed by solvent evaporation or extraction. The choice of the droplet stabilizer during the emulsion step is critical for the dimensions and the surface characteristics of the nanoparticles (NPs). In the present work, a recently described ionic amphiphilic chitosan derivative, chitosan oleate salt (CS-OA), was proposed for the first time to prepare PLGA NPs. A full factorial design was used to understand the effect of some formulation and preparation parameters on the NP dimensions and on encapsulation efficiency (EE%) of Nile red, used as a tracer. On the basis of the DoE study, curcumin loaded NPs were prepared, having 329 ± 42 nm dimensions and 68.75% EE%. The presence of a chitosan coating at the surface was confirmed by positive zeta potential and resulted in mucoadhesion behavior. The expected improvement of the interaction of the chitosan surface modified nanoparticles with cell membrane surface was confirmed in Caco-2 cell culture by the internalization of the loaded curcumin.

## 1. Introduction

Poly-lactic-glycolic acid (PLGA) is one of the most widely used biodegradable polymers in nanoparticle (NP) formulations, thanks to possible modulation of biodegradation rate by means of the choice of suitable PLGA grades and thanks to good regulatory position, as they are well accepted by FDA and EMA [1,2].

Most of the methods of PLGA NPs preparation involve the passage through the emulsification of a PLGA organic solution in water followed by solvent evaporation or extraction. The choice of the droplet stabilizer during the emulsion step is critical for the dimensions and the surface characteristics of the NPs. The most used stabilizer in the literature is PVA [3]. Few other polymers were demonstrated to be useful to stabilize nanoemulsions, such as carbomer and poloxamer, while other macromolecules, such as cellulose derivatives and gelatin, resulted in NPs with acceptable dimensions only when used in association with PVA [4]. In most cases the zeta potential resulted negative, with the only exception of the association between PVA and the cationic gelatin A [4].

A growing interest can be seen in recent years in the literature for PLGA NPs coating to modify the surface to cationic charge. Positively charged NPs can in fact more efficiently interact with negatively charged cellular membranes triggering cell uptake [5,6,7,8]. From this perspective, the coating with chitosan is in many cases a first choice for its low cost, good biocompatibility, and interesting biological properties such as mucoadhesion [9], that in turn can support the efficiency of nanosystems for mucosal vaccination [10,11]. Moreover, chitosan coated PLGA NPs are described as useful tools to improve transfection in nucleic acids delivery [12,13]. An early systematic study was performed to assess the possible employment of chitosan as stabilizing agent in the preparation of NPs, with and without PVA. While chitosan alone resulted not suitable to stabilize the particles, PVA-chitosan blends led to NPs of low dimensions and positive surface charge [14]. A further approach involves the preparation of PVA-stabilized NPs coated in a second step by chitosan electrostatic adsorption [6,15,16].

Polymeric surfactants present peculiar efficiency in the stabilization of nanoemulsions, due to the multiple contact points of the hydrophobic moieties with the o/w interface and steric effect of hydrated hydrophilic chains [17]. If a derivative of a bioactive polymer is used, some of the polymer properties can be maintained by the nanoemulsion. Recently, some evidence was given of the ability of a new amphiphilic chitosan salt, chitosan oleate CS-OA [18], obtained by ionic interaction between chitosan and oleic acid, to stabilize an essential oil nanoemulsion [19]. It was observed that a low energy method could be combined with the self-assembling behavior of chitosan after electrostatic interaction with the hydrophobic moieties of oleic acid. Nanoemulsions of dimensions in the nanometric range were obtained, depending on chitosan concentration and resulted physically stable for at least three months. The zeta potential of the nanoemulsion confirmed that the chitosan derivative was adsorbed at the droplet interface thanks to affinity of oleic chains for hydrophobic phase, while chitosan backbone resulted arranged towards the aqueous medium [19]. Chitosan oleate maintained in this case an antimicrobial effect, in accordance with literature findings [20,21]. Nanoemulsions based on alpha tocopherol and stabilized by means of chitosan oleate salt were studied on fibroblast and keratinocyte cell lines, on ex vivo human biopsies, and in vivo on a rat burn model, showing the biological effects of both chitosan and oleic acid on the wound healing promotion [22,23].

In the present work, the physical stabilization of nanoemulsions by chitosan oleate was for the first time exploited in the preparation of chitosan coated PLGA NPs. The peculiar arrangement of the hydrophobically modified polymer at the oil to water interface around the droplets should in fact result in the exposure of polysaccharide chains towards the aqueous environment so that solvent removal would lead to the occurrence of PLGA NPs coated with a chitosan shell. In the perspective of a Quality by Design-based pharmaceutical development, the DoE approach is more and more encouraged. In this frame, screening factorial designs are a useful instrument to study the relevance of formulation, preparation and assessment variables on product properties [24,25]. A screening full factorial design was here used to understand the effect of the ratio between chitosan and oleic acid, of chitosan concentration and of stirring rate on the NP dimensions and on encapsulation efficiency of Nile red used as a tracer. To confirm the interaction with the biological substrates, curcumin loaded NPs were prepared and tested for mucoadhesion properties and internalization in Caco-2 substrates. Curcumin was chosen as a hydrophobic model molecule for its largely studied behavior, and the possibility to compare the results obtained with the proposed new CS-OA based systems with those obtained with NPs previously described in the literature [26,27,28]. 

## 2. Results and Discussion

**Figure 1:**
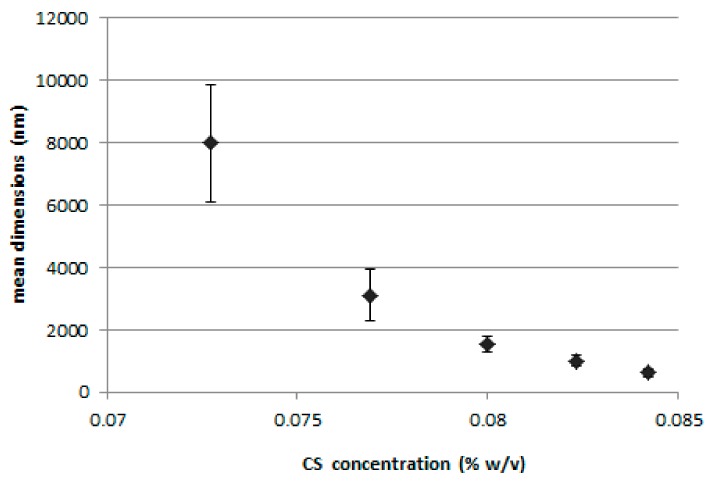
Dimensions of the dispersion (mean ± SD; *n* = 3) for different chitosan (CS) final concentrations.

Figure 1 shows the results of the preliminary study performed to assess the relationship between the amount of CS-OA used during the preparation of the nanoparticles and their final dimensions. The CS-OA was obtained in situ by electrostatic interaction between the polysaccharide and the oleic acid, at a 1:1 stoichiometric ratio. Increasing polymer dispersion volumes were added during the emulsification step to stabilize the dispersion of PLGA ethyl acetate solution in water. Final CS concentrations resulted in a range between 725 and 840 µg/mL, while the amount of ethyl acetate PLGA solution and polymer concentration were maintained constant. The three samples prepared with the lowest volumes of CS-OA dispersion corresponded to the highest mean particle size values, of 7.99 (±1.87) µm, 3.11 (±0.82) µm, and 1.54 (±0.23) µm. These samples were characterized by laser diffraction apparatus and showed a clear decrease of the percentage of dispersion exceeding the nanometer dimensions with the increase in chitosan derivative volume. By further increasing the CS-OA amount, the resulting particulate dimensions decreased below 1000 nm. The comparison between the two series of data can of course be considered just indicative, as the measurements of the dimensions are obtained with two different apparatus, but nevertheless suggested the use of CS-OA concentrations in a higher range. To better assess the relevance of CS-OA amount, CS to OA ratio, and of a preparative parameter such as ULTRA-TURRAX speed, a DoE-based evaluation was performed on Nile red loaded NPs.

### 2.1. Relevance of Formulation and Preparation Parameters on Nile Red Loaded NPs through DoE Study

In this phase of the development study, the volume of CS-OA dispersion was maintained constant (at 10 mL) and Nile red-loaded NPs were prepared by checking the possible further reduction of the dimensions by increasing the stirring rate and the initial chitosan concentration. The relevance of hydrophobic modification level was evaluated by comparing CS-OA at 1:0.2 and at 1:1 chitosan:oleic acid molar ratio, in a range between a low substitution and the maximum stoichiometric ratio.

Stirring rate range was from 13,500 rpm, slightly higher than the stirring rate used in Phase 1, to 20,500 rpm, the highest rate for the used apparatus.

Chitosan concentration was studied in the range 0.1% (*w*/*v*) slightly higher than the maximum one considered in Phase 1, and 0.2% (*w*/*v*), that in a previous work allowed the authors to obtain nanoemulsions of a few hundred nanometers in size [19].

Nile red was encapsulated in the NPs as a fluorescent tracer, for its hydrophobic character, compatible with loading in PLGA core of the NPs. A full factorial was designed and both the dimensions of the PLGA NPs and the encapsulation efficiency of Nile red were evaluated as a response.

Table 1 reports the data obtained for the eight samples and for the three central points of the Full Factorial 2^3^ experimental design. 

All the data come from analysis replicates (whose variability is described by the standard deviation values) while the replicates necessary for the variability evaluation of the model were performed on the central point. Each set of data was evaluated for standardized skewness and standardized kurtosis and the values resulted within the range expected for data from a normal distribution.

Apart from the two samples with the lowest concentration of chitosan and the lowest ratio between chitosan and oleic acid, whose dimensions were as high as 1830 nm and 1186 nm for 13,500 and 20,500 rpm stirring rate, respectively, in all other cases the average size is maintained in the range of NP systems. All the samples analyzed have an encapsulation efficiency higher than 65%, and as high as 89.58% in the case of the sample prepared with chitosan concentration 0.2% (*w*/*v*), CS-OA ratio 1:1 and stirring rate 13,500 rpm.

The model used to fit the data of NP dimensions is as follows.

dimensions = 834,636 − 202,875*A − 248,125*B − 48,125*C + 190,375*A*B + 110,875*A*C + 57,125*B*C

**Figure 2:**
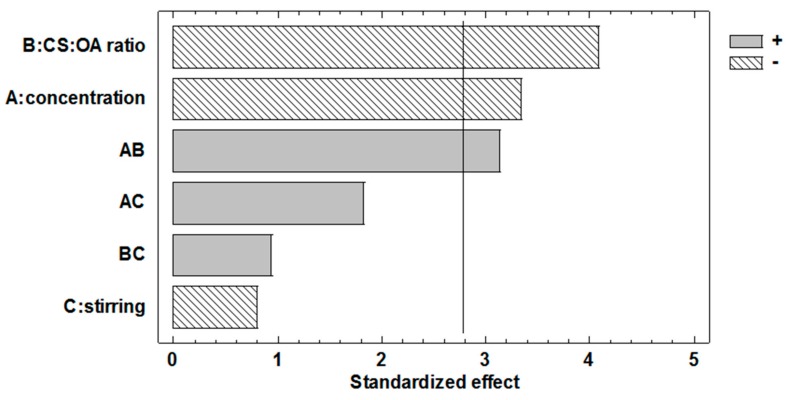
Pareto chart obtained from the statistical analysis of the 2^3^ full factorial design and illustrating the effects of the three factors considered and of their interactions on poly-lactic-glycolic acid (PLGA) nanoparticle (NP) dimensions.

The Pareto chart for NP dimensions (Figure 2) and the ANOVA table (Table 2), show that both chitosan concentration and stoichiometric ratio between chitosan (CS) and hydrophobic agent (OA) significantly influence the response. Both these effects have a negative sign, so that the higher concentration (0.2% *w*/*v*) and the higher CS:OA ratio (1:1) contribute to decrease the average size of the particles. The effect of concentration factor confirms the trend observed in the preliminary part of the study (Figure 1). The effect of hydrophobic modification seems to confirm its importance for the anchoring of CS-OA at oil to water interface. This is in line with literature data that found poor stabilization of the emulsion with chitosan alone [14]. It is conceivable however that the theoretical stoichiometry between chitosan and oleic acid does not correspond to the final composition of the modified polymer, as it is likely that not all the amino groups of the polymer are involved in the interaction with the fatty acid. 

**Figure 3:**
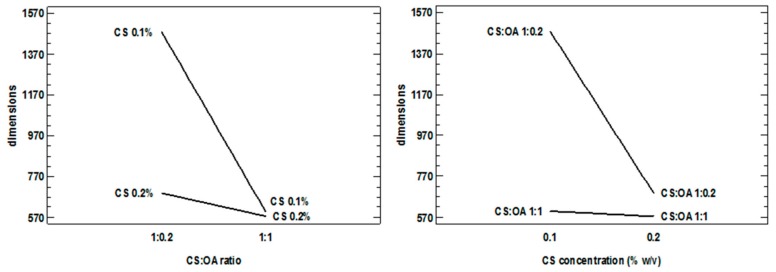
Interaction plots obtained from the statistical analysis of the 2^3^ full factorial design and illustrating the interactions between the factors concentration (CS % *w*/*v*) and chitosan:oleic acid (CS:OA) ratio.

The graph of interaction between concentration and polymer ratio and hydrophobic agent shows a very clear interdependence, confirmed by the statistics that see this significant interaction (Figure 3). However, each of the factors assumes a marked importance in decreasing the particle size when the other factor is at the lowest level, and the stability of the dispersion is probably more critical.

The Pareto chart shows that the mechanical agitation during preparation is not significant, contrary to what could be expected. 

The model used in the case of EE% as response is as follows.

EE% = 77.6151 + 4.94963*A + 4.27651*B – 2.54119*C − 0.332076*A*B + 1.38938*A*C − 1.53579*B*C

In this case a reduced model obtained by exclusion of the not significant interaction with the lowest coefficient was chosen.

**Figure 4:**
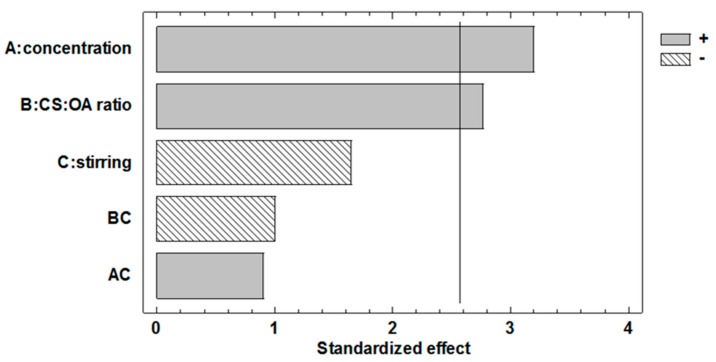
Pareto chart obtained from the statistical analysis of the 23 full factorial design illustrating the effects of the three factors considered and their interactions on Nile red encapsulation efficiency (EE%) in PLGA NP.

Regarding the effect of the factors on the efficiency of encapsulation, the Pareto graph (Figure 4) and the corresponding ANOVA (Table 3), suggest that also in the case of EE% response, the chitosan concentration and the molar ratio between the polymer and the hydrophobic agent have a significant and positive effect. This is in line with their relevance on nanoemulsion stabilization during nanoparticle preparation as it seemed conceivable that quick and efficient coating of the droplets should help the retention of the marker inside the NPs.

### 2.2. Curcumin Loaded NPs

Considering the results of the DoE study on critical parameters, the following conditions were chosen to prepare chitosan coated PLGA NPs: chitosan concentration 0.2% (*w*/*v*), chitosan:oleic acid ratio 1:1, and 24,000 rpm ULTRA-TURRAX stirring rate. As reported in Table 1, however, the dimensions of the NPs were even in this case higher than 500 nm. To further reduce the dimensions, acetone 5% *v*/*v* was added together with the hydrophobic phase. It is reported in the literature that acetone, as water miscible solvent, during addition to aqueous environment is subject to quick diffusion. This effect lowers the interfacial tension and makes the dispersion of the droplets finer, resulting in turn in small NP dimensions [29]. 

According to this mechanism, the dimensions of the CS-OA stabilized NPs were reduced, making possible a direct comparison of CS-OA-stabilized NPs with samples obtained by using PVA to stabilize the nanoemulsion, according to the procedure described in the literature [3]. The comparison of the two nanosystems, prepared with CS-OA and with PVA, both unloaded and loaded with curcumin, was performed by considering dimensions, polydispersion index, and zeta potential (Table 4). EE% values are also given in Table 4 in the case of curcumin loaded systems. 

Considering the dimensions, the NPs obtained by using CS-OA as stabilizer, both unloaded (CS-OA PLGA) and curcumin loaded (Cur-CS-OA PLGA), were in a range of few hundred nanometers, comparable to the results obtained by using PVA. As the dimensions, at the same process conditions, are influenced mainly by surfactant effect of the polymers during emulsification, these results confirm the efficiency of CS-OA as amphiphilic polymer in emulsion stabilization. EE% was slightly higher for PVA NPs (~82%) with respect to CS-OA NPs (~70%). EE% differences can be here explained by a different arrangement of curcumin at the NP surface. This could represent also a possible explanation for the different zeta potential values observed for unloaded and curcumin loaded NPs. Zeta potential is clearly positive for both the unloaded and curcumin loaded CS-OA NPs, confirming the presence of a chitosan shell at the NP surface.

In both the systems the NPs showed EE% values quite high, in line with the hydrophobic nature of curcumin and its good affinity for the PLGA core. On the basis of EE% values, the curcumin colloidal concentration was calculated, ranging between approximately 82 µg/mL in the case of Cur-CS-OA PLGA and almost 100 µg/mL in the case of Cur-PVA PLGA. In both cases a clear improvement of concentration was observed with respect to curcumin solubility, that literature reports as low as 11 ng/mL [30].

**Figure 5:**
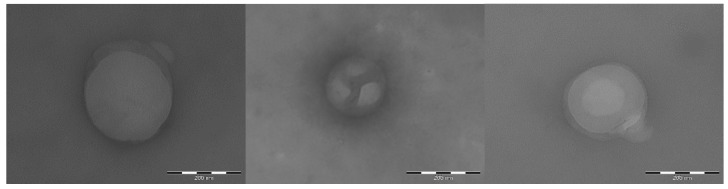
Representative TEM images of CS-OA PLGA nanoparticles. Bars: 200 nm.

Figure 5 reports some representative TEM images of CS-OA PLGA NPs. The NPs observed in TEM analysis, like those reported in Figure 5, showed dimensions of few hundred nanometers, in accordance with the results of PCS analysis shown in Table 4, although the two methods differ by physical principle, sampling, and information obtained. From the morphological point of view, TEM images show nonaggregated, spherical NPs. In particular, for those CS-OA PLGA NPs in which the chitosan coating was partially interrupted, like in the images selected for Figure 5, it was possible to appreciate a less dense homogeneous core corresponding to the PLGA inner matrix, and the presence of a more dense surface outer layer conceivably represented by the chitosan coating.

#### 2.2.1. Curcumin Loaded NPs Mucoadhesion Behavior

**Figure 6:**
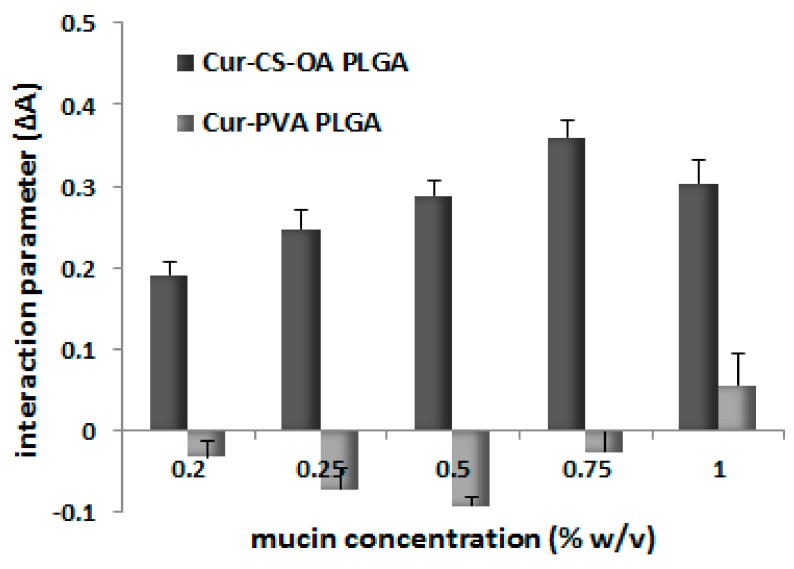
Interaction parameter (mean ± SD, *n* = 4) as a function of mucin concentration for Cur-CS-OA and Cur-PVA NPs. The differences between the two samples were statistically significant at all the mucin concentrations (Mann–Whitney, *p* < 0.05).

The mucoadhesion behavior of the NPs is illustrated in Figure 6. In particular, Figure 6 shows the results of the test performed in vitro based on the interaction between the NPs and a commercial mucin dispersion. The difference in absorption (ΔA) between the measured absorbance A and the theoretical one (Atheor), indicated as interaction parameter, was proposed in the literature [31] and gives a measure of the interaction between the mucin and the NPs. When no interaction takes place, ΔA = 0 while values of ΔA > 0 indicate a strong interaction between the mucin and the micelles. This approach was previously validated for NP systems [32] by correlation of the positive values of interaction parameter with positive interaction between NP surface and mucin assessed with different other in vitro and ex vivo tests. A positive mucoadhesive interaction clearly occurs here with Cur-CS-OA PLGA chitosan-coated NPs while it is not visible for uncoated ones, as it could be expected for the chitosan presence on the NP surface thanks to its well-known mucoadhesive behavior.

#### 2.2.2. Curcumin Loaded NPs. Interaction with Caco-2 Cell Lines

**Figure 7:**
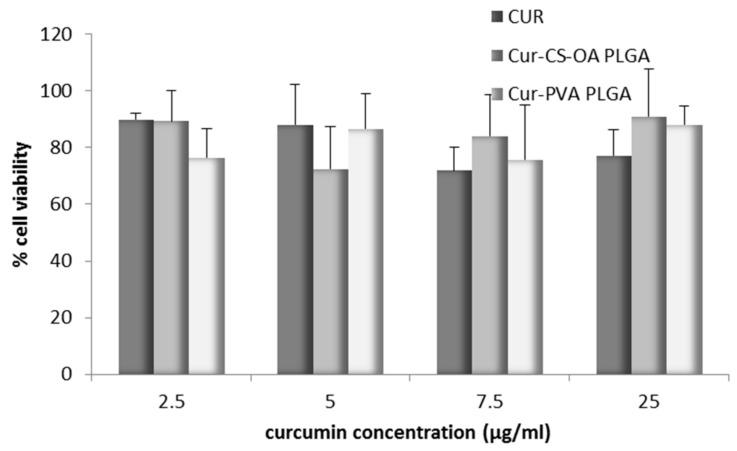
Biocompatibility with Caco-2 cell lines for the samples Cur-CS-OA PLGA, Cur-PVA PLGA, and free curcumin at different curcumin concentrations, as % of cell vitality with respect to the controls after 24 h (mean ± SD, *n* = 8).

Figure 7 confirms the good biocompatibility of the curcumin loaded samples in a range of curcumin concentrations up to 25 µg/mL. In all cases cell viability was around 80% of the controls, without differences between the free curcumin in DMSO and the two NP samples. This result is in line with what was observed by Beloqui et al. [27] that on the basis of cytotoxicity studies, treated Caco-2 cells with NPs combining poly(lactide-co-glycolide) acid (PLGA) and a polymethacrylate polymer with curcumin concentration of 75 µg/mL. Therefore, Caco-2 cells seem less sensitive to curcumin effect than other cell lines such as A2780 CP or MDA-MB-231 studied by Yallapu et al. [33] or some tumoral cells studied by other authors [26,34] that found 25 µM curcumin both free and loaded in NPs strongly cytotoxic, close in many cases to DL_50_ values. 

**Figure 8:**
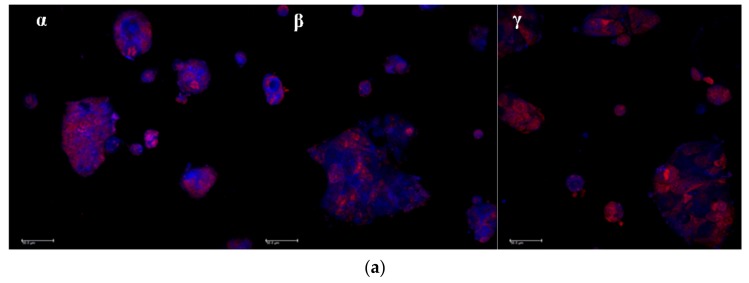

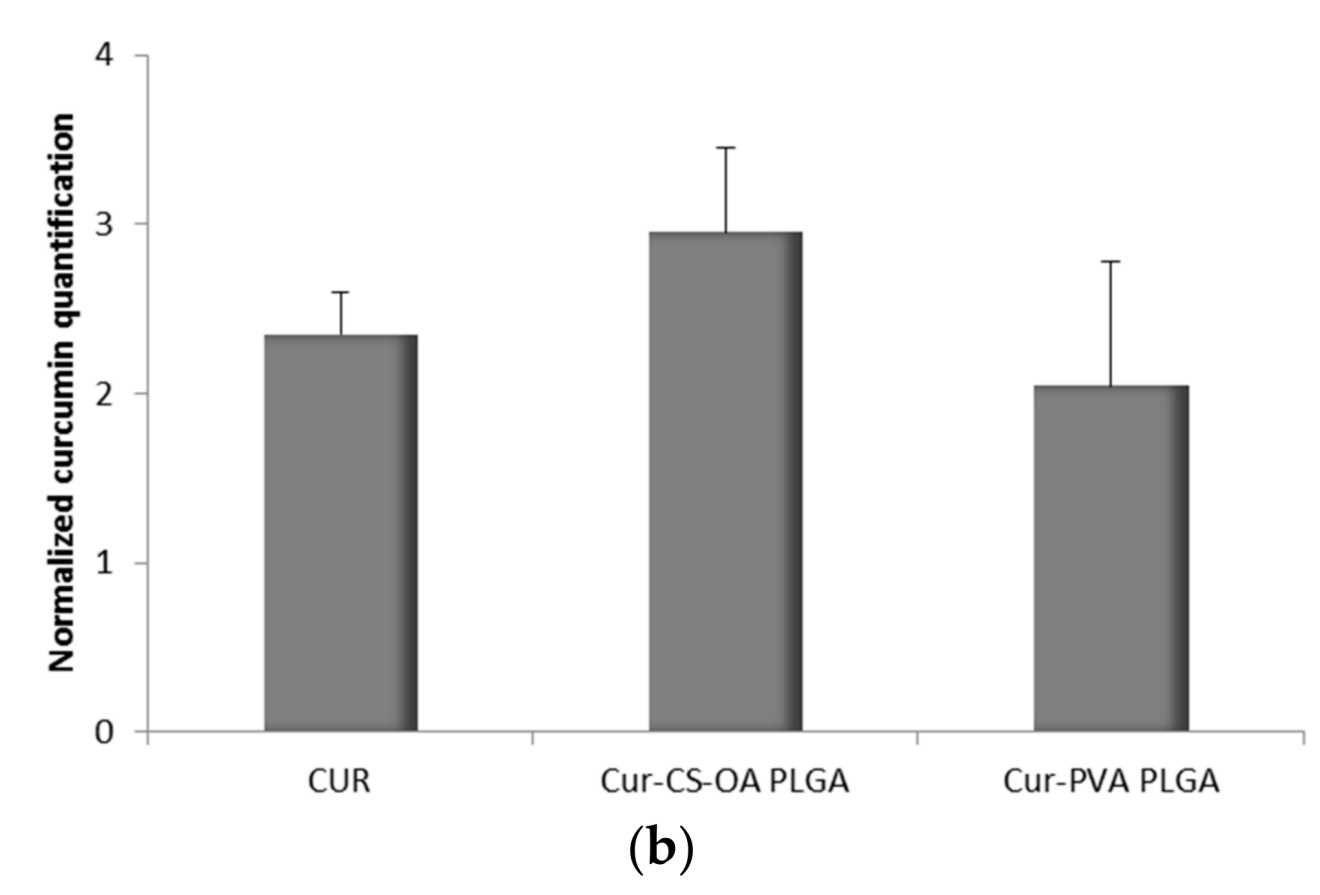
(**a**) Representative CLSM photomicrographs of Caco-2 cell substrates grown after 48 h on microscope slides and treated 24 h with the samples. (α) Free curcumin; (β) Cur-CS-OA PLGA; (γ) Cur-PVA-PLGA. Bar: 50 µm; (**b**) CLSM fluorescence quantification, obtained by image analysis, of the amount of curcumin associated to the cells grown on microscope slides (mean ± SD; *n* = 3).

The association of curcumin with Caco-2 cells grown 48 h on microscope slides is illustrated in Figure 8a,b. The pictures in Figure 8a are representative images of clusters of cells whose nuclei are stained in red by propidium iodide, while the blue staining indicates the curcumin presence in cytoplasm. In Figure 8b, the curcumin quantified in the cells by image analysis elaboration of the Confocal Laser Scanning Microscopy (CLSM) fluorescence signal, is illustrated for each sample. Cur-CS-OA PLGA sample shows strong positive association to the cells. The good internalization of free curcumin inside the cells is probably determined by its hydrophobic character, that allows easy passage of the cell membranes of the molecule once it is in solution.

**Figure 9:**
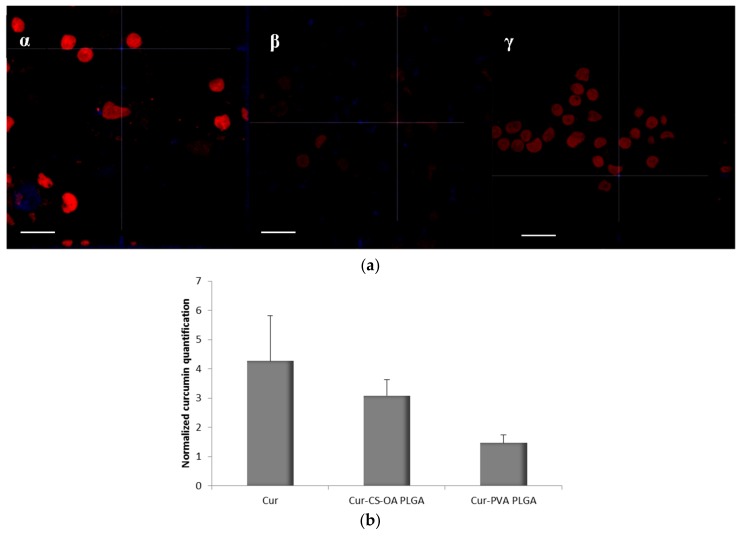
(**a**) Representative CLSM photomicrographs with z planes of Caco-2 cell substrates grown on transwell membranes, and treated for 3 h with the samples. (α) Free curcumin; (β) Cur-CS-OA PLGA; (γ) Cur-PVA PLGA. Bar: 20 µm; (**b**) CLSM fluorescence quantification, obtained by image analysis, of the amount of curcumin associated to the Caco-2 cells grown on transwell membrane (mean ± SD; *n* = 3).

Figure 9a,b refers to cells grown on transwell membranes. In this experimental set the Caco-2 cells reach full polarization and are able to express tight junctions. Also in this case, curcumin can be seen inside the cells, as indicated by the blue staining around the nuclei and along the Z axis. Figure 9b shows the results of fluorescence quantification. CS-OA coated NPs seem to be responsible of good internalization in cells, slightly lower than that of free curcumin, but higher than that of PVA NPs. 

**Figure 10:**
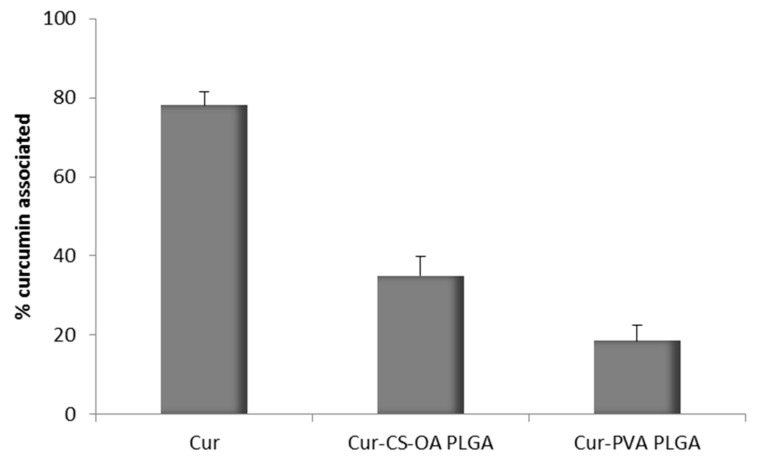
Percentage of curcumin associated to the Caco-2 cell substrates (mean ± SD; *n* = 8) detected by fluorescence analysis. Statistically significant differences (one-way ANOVA, post-hoc Fisher’s test, *p* < 0.05): Cur vs. Cur-CS-OA PLGA, Cur vs. Cur-PVA PLGA, Cur-CS-OA PLGA, and vs. Cur-PVA PLGA.

In no case curcumin could be quantified in the acceptor compartment, in line also in this case with other author results, that did not find quantifiable Papp values for curcumin through Caco-2 cells [27]. However, it was possible to measure the curcumin concentration by fluorescence analysis in the apical compartment at the end of the 3 h test. On the basis of these results the amount of curcumin associated with Caco-2 substrate treated with the samples was calculated by difference. The results are illustrated in Figure 10 and support what previously observed by CLSM pictures. In the case of Cur-CS-OA PLGA sample about 40% of the curcumin put in contact with the cell substrates seems associated with the cell layers. Statistically significant differences can be seen between Cur-CS-OA PLGA and both curcumin and Cur-PVA PLGA (one-way ANOVA, post-hoc Fisher’s test). 

**Figure 11:**
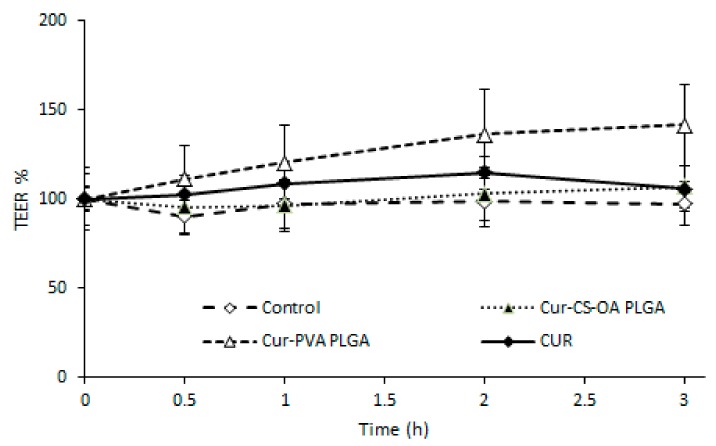
Transepithelial/endothelial electrical resistance (TEER) % profiles of Caco-2 substrates on transwell membranes after contact with Cur-CS-OA PLGA, with Cur-PVA PLGA, and with free curcumin (mean ± SD; *n* = 4).

The transepithelial electrical resistance (TEER) profiles during the three hours of contact of the samples with the substrates (Figure 11) show that no decrease occurred, even with the CS-OA-coated sample, indicating that the presence of the chitosan layer around the NPs does not influence in this case the tight junction structure. This result was not expected, as there are many example in the literature reporting that chitosan-based NPs open tight junctions. The result obtained in this case could be due to a concentration effect, as previously observed with chitosan coated PLGA nanoparticles on Calu3 [35], or to tight association of chitosan with the NPs in line with previous studies on chitosan palmitate polymeric micelles in which it was put in evidence that among different substitution grades, only the less substituted and more hydrophilic derivative was able to maintain the capability to decrease TEER values [36].

## 3. Materials and Methods 

### 3.1. Materials

The following materials were used, chitosan (CS) was obtained as HCl salt from low molecular weight (LMW, chitosan base, deacetylation degree 80% (Sigma-Aldrich, Milan, Italy), by addition of HCl 0.5 N to chitosan until complete dissolution, dialysis in bidistilled water for 24 h and freeze-drying (HetoDrywinner, Analitica de Mori, Milan, Italy). Oleic acid was from Fluka (Milan, Italy). PLGA Resomer RG 503H, Low MW grade poly-vinyl-alcohol (PVA), Nile red and curcumin were all from Sigma-Aldrich. Acetone, acetic acid, sodium acetate, and sodium chloride were acquired from Carlo Erba (Milan, Italy).

### 3.2. Preparation of the Chitosan Coated NPs

Chitosan oleate (CS-OA) was obtained in situ, as previously described [19,22], by self-assembling during the preparation of the samples. Briefly, oleic acid dissolved in acetone was added to chitosan HCl solution at either 0.1 or 0.2% *w*/*v* and acetone was removed under stirring for about 20 min. The ratio between chitosan and oleic acid was calculated as molar ratio taking into account the molecular weight of glucosamine unit and the theoretical free amino groups of chitosan. Considering the 80% deacetylation degree in accordance with the used chitosan grade, a 1:1 ratio corresponded to 1.4 mg of oleic acid per each mg of chitosan.

In a first step a 0.1% (*w*/*v*) of chitosan concentration and 1:1 stoichiometric ratio with oleic acid were used. 2.5 mL of ethyl acetate solution containing 12 mg PLGA were added to 3 mL distilled water. Emulsification started at 9500 rpm by means of ULTRA-TURRAX T25 (Janke & Kunkel, IKA^®^ Labortechnik, Germany) equipped with 10 mm probe (S25 N-10 G) and after 5 min different volumes of CS-OA dispersion, between 8 and 16 mL, were added. After 10 min, ethyl acetate was removed under stirring at 40 °C for about 45 min. The weight lost during evaporation was assessed and the initial volume reconstituted with distilled water.

### 3.3. Dimensional and Zeta Potential Characterization of Dispersed Phase

The particle size of the dispersed phase was measured by laser diffraction, Microtrac (Microtrac^®^ SRA 150 ASVR, Honeywell, Phoenix, AZ, USA) for samples exceeding the nanometer range. For the samples in the nanometric range the dimensions and the Polydispersity Index (PI) were measured by Photon Correlation Spectroscopy (PCS) (N5 Submicron Particle Size Analyzer Beckman Coulter, Milan, Italy). PCS analysis were performed at 90° detection angle after dilution in 0.22 µm filtered bidistilled water. PI indicates the width of the size distribution ranging between 0 (monodispersity) and 1. At least three replicates were performed. Zeta potential measurements were performed by means of a Zetasizer^®^ Nanoseries (Malvern Instruments Ltd., Worcestershire, UK). Three measurements were performed for each sample.

For TEM analysis the samples have been layered on 300 mesh cupper grids. The images have been obtained with a JEOL JEM1200EX II apparatus (JEOL Ltd., Tokyo, Japan).

### 3.4. Nile Red Loaded NPs

For Nile red-loaded NPs, a full factorial 2^3^ design was used to study the following formulation and preparation factors (independent variables) each of them set at two levels as hereafter indicated, chitosan concentration 0.1% *w*/*v* (−1) and 0.2% *w*/*v* (+1), chitosan to oleic acid ratio 1:0.2 (−1) and 1:1 (+1), and ULTRA-TURRAX speed at 13,500 rpm (−1) and 20,500 rpm (+1). In this study, 100 µL of 1 mg/mL Nile red in ethyl acetate were added together with 2.5 mL of 4.8 mg/mL ethyl acetate PLGA solution to 10 mL of chitosan oleate aqueous dispersion prepared as previously described. Two response (dependent) variables, that is nanoparticle dimensions and Nile red encapsulation efficiency, were considered. According with the 2^3^ full factorial design, eight experiments were performed. Moreover, one central point was added and replicated three times. All the experiments were performed in a randomized sequence. 

#### Nile Red Encapsulation Efficiency (EE%) Evaluation

Nile red was quantified by UV–Vis detection (Perkin Elmer Instrument Lambda 25 UV–Vis Spectrometer, Monza, Italy) in CH_3_CN and acetate buffer 0.1 M pH 4.0, 80:20 mixture, where it was previously verified that all the NP components could be solubilized. Absorbance was read at 552 nm, where the maximum of absorbance was found in the solvent used. The encapsulation efficiency (EE%) was calculated as the percentage ratio between the amount of the tracer quantified in the NPs and the amount added to the formulation. To determine the EE% the samples, after centrifugation 10 min at 6000 rpm to remove by precipitation the amount not encapsulated, the supernatant was diluted in CH_3_CN and acetate buffer mixture and spectrophotometrically read.

### 3.5. Preparation of Curcumin Loaded NPs

**Figure 12:**
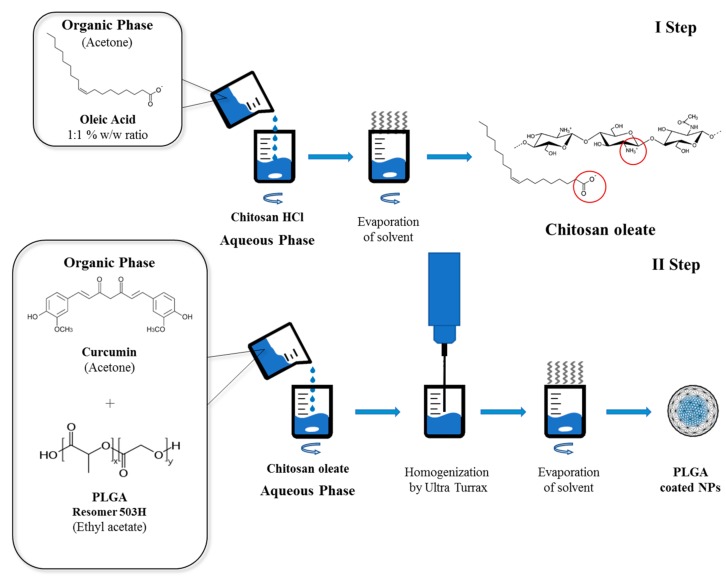
Schematic representation of the preparation method of chitosan-coated NPs.

Curcumin loaded NPs were obtained with the same solvent evaporation procedure (Figure 12) described above for Nile red loaded NPs. 1.2 mg of curcumin dissolved in 500 µL of acetone were added together with 2.5 mL of 4.8 mg/mL ethyl acetate PLGA solution to 10 mL of chitosan oleate aqueous dispersion prepared as previously described, to obtain a final concentration of 120 µg/mL. In this case, to stabilize the nanoemulsion, the CS-OA dispersion obtained as previously described from 0.2% *w*/*v* chitosan and 1:1 chitosan and oleic acid ratio was used. For comparison purposes, NPs stabilized with 2% *w*/*v* PVA solution according to the literature [37] were prepared. In both cases, ULTRA-TURRAX homogenization was performed at 20,500 rpm. 

#### Curcumin Quantification and EE% Evaluation

Curcumin was quantified by UV–Vis detection in CH_3_CN and acetate buffer 0.1 M pH 4.0, 80:20 mixture. Absorbance was read at 431 nm wavelength. To determine the EE% of the samples (after centrifugation at 10 min at 6000 rpm to remove by precipitation the amount not encapsulated) the supernatant was diluted in CH_3_CN and acetate buffer mixture and spectrophotometrically read.

### 3.6. Mucoadhesion Evaluation

For the in vitro test, different concentrations, ranging between 0.1% and 1.0% *w*/*v* of mucin type I (Sigma-Aldrich, Milan, Italy), were prepared and put in contact with 200 µL of NP sample for 2 min. Turbidimetric measurements were carried out using a spectrophotometer (UV–Vis Lamba 25, Perkin Elmer, Milan, Italy) at λ = 500 nm. The mixtures of samples and mucin at increasing concentrations, were compared with the dispersions containing the same concentration of mucin as in the mixture [32]. The turbidimetry values were also elaborated according to the method described in the literature [31]: the effective absorbance (A) was that of the mixture of mucin and NPs. The theoretical absorbance (Atheor) was calculated by adding the absorbance of NPs and that of mucin dispersion at the same concentration. The interaction parameter was the difference in absorption (ΔA) between the measured absorbance and the theoretical one. 

### 3.7. Caco-2 Biocompatibility Test

Caco-2 cell culture was grown in a polystyrene flask in complete culture medium consisting of DMEM added with 1% *v*/*v* of a penicillin–streptomycin–amphotericin 100× solution (pen/strep/amph; Euroclone, Milan, Italy), 10% *v*/*v* of inactivated fetal FBS bovine serum (Fetal Bovine Serum; Euroclone, Milan, Italy) and 1% *v*/*v* of nonessential amino acids 100× (Mem Nonessential Amino Acid Solution 100×; Sigma-Aldrich, Milan, Italy). The cells were grown in an incubator (CO_2_ Incubator, PBI International, Milan, Italy) at 37 °C, in a humidified atmosphere, containing 5% of CO_2_.

For the cytotoxicity test, Caco-2 cells were seeded (0.35 × 10^5^ cells per well) in 96-well plates (Cellstar 96 Well Culture Plate, Greiner Bio-One, Frickenhausen, Germany) with an area of 0.36 cm^2^. After seeding, the plates were placed in the incubator for 24 h, then the cells were washed with 100 μL of PBS, and the samples were added.

NPs loaded with curcumin were tested on cell lines to assess biocompatibility at final curcumin concentration ranging from 2.5 to 25 µg/mL concentration. After 24 h of contact with the samples, a MTT test was performed. The MTT test is based on conversion of tetrazolium salt (3-(4,5-dimetiltiazol-2-)-2,5-diphenyltetrazolium bromide) to formazan by mitochondrial dehydrogenases of vital cells [38]. Briefly, 50 μL of 2.5 mg/mL MTT solution (Sigma-Aldrich, Milan, Italy) in HBSS (Hank’s Buffered Salt Solution) pH 7.4 was put in contact with each cell substrate for 3 h. After removing the reagent, the substrates were washed with 200 μL of PBS. Then 100 μL of DMSO was put in each well. The absorbance was read at 570 nm by means of an ELISA plate reader (Imark Absorbance Reader, Biorad, Milan, Italy). Cell viability was calculated as percentage ratio between the absorbance of each sample and the absorbance of controls (cell substrates in growth medium).

### 3.8. Cell Uptake Studies

Cell uptake was evaluated by means of confocal laser scanning microscopy (CLSM). This was performed both on cells grown 48 h on slides and on cells grown on transwell membranes. In the first case the cells were seeded in 24-well plates. At the bottom of each well was a 13 mm diameter slide (Coverglass Borosilicate, VWR International, Milan, Italy) on which the cells were made to adhere and grow for 24 h. The samples were added and left in contact with the cells another 24 h, after which the cell substrates were washed with 500 µL of PBS and fixed with a 3% *v*/*v* glutaraldehyde solution in PBS for one hour at 4 °C. The glutaraldehyde was removed and the wells were washed twice with 500 µL of PBS.

Cells were also seeded on transwell membranes (0.4 µm pores, 1.13 cm^2^ surface, Cellstar^®^ permeable support tissue culture plate, Greiner bio one^®^, Milano, Italy) and grown 20 ± 1 days until complete confluence. Monolayer integrity was verified by checking TEER values. The basolateral compartment was filled with 1.5 mL of DMEM without Phenol red to avoid fluorescence interferences. Five-hundred microliters of each sample was diluted in DMEM at a final curcumin concentration of 24 µg/mL and placed in donor chamber. After 3 h curcumin was quantified by fluorescence analysis in the apical and basolateral compartment by means of a microplate reader (Plate reader, Synergy HT, 14041014) at 485 nm excitation and 528 nm emission. The cell substrates were washed with PBS, treated 10 min with paraformaldehyde and washed twice with PBS.

Nuclei were stained just before the microscope analysis with 150 µL of propidium iodide (PI) 20 µg/mL in PBS for 5 min in the dark. The PI was then removed and the cells washed with 200 μL of PBS. The slides were examined using a laser scanning confocal microscope (CLSM, Leica TCS SP5II, Leica Microsystems CMS GmbH, Milan, Italy), which allows visualization of fluorescence of propidium iodide (λex = 520 nm and λem = 630 nm) and of curcumin (λex = 440 nm and λem = 520 nm). The fluorescence was quantified on all the microphotographs collected (at least 3 for each sample) by means of an Image analysis program (ImageJ 1.46r, NIH, Bethesda, MD, USA). The blue intensity of curcumin was normalized per each image by the red fluorescence of nuclei.

### 3.9. Statistical Analysis

Statistical evaluations were performed by means of Statgraphics 5.0, Statistical Graphics Corporation, MD, USA. Differences were determined according to one-way ANOVA and were considered significant at *p* < 0.05. The same statistical package was used to analyze the results of the full factorial design.

## 4. Conclusions

The results obtained in this study appear promising for the application of an ionic chitosan derivative, such as the chitosan oleate salt, in the easy preparation of nanoparticles with hydrophobic cores that are surface modified with a hydrophilic polysaccharide corona. The high zeta potential values confirm the presence of chitosan at the nanoparticle surface. Chitosan coating can be advantageous for its well-known biological properties and for increased possibilities of further derivatization. Efficiency of the chitosan derivative in PLGA NP preparation resulted comparable to that of PVA. Clear improvement of mucoadhesion behavior has been obtained for CS-OA-based NPs. Further work will be necessary to better understand the mechanism of association between chitosan and oleic acid and its final stoichiometry. Also, the interaction of the nanoparticles with biological substrates and the possible improvement of absorption enhancement properties would deserve better investigation, although the preliminary results here obtained confirm a positive cell internalization of the surface modified nanoparticles, likely due to the interaction of cationic chitosan with the anionic cell membrane. Considering the good potentiality of the PLGA core in the loading of hydrophobic drugs and the mucoadhesion and penetration enhancement properties of the chitosan shell, applications in the delivery of poorly soluble and poorly absorbable drugs can be envisaged.

In a more general perspective, the use of amphiphilic derivatives of bioactive polymers, like chitosan, as nanoemulsion stabilizers in solvent evaporation methods can be proposed as a useful approach for surface modification of nanoparticles with bioactive polymeric shells.

## Figures and Tables

**Table 1:** Dimensions and encapsulation efficiency (EE%) (mean ± SD; *n* = 4) of the samples prepared according to the 2^3^ full factorial experimental design taking into account, as factors, the chitosan (CS) concentration 0.1–0.2% (*w*/*v*), the stoichiometric ratio chitosan: oleic acid (CS-OA) (1:0.2–1:1), and the ULTRA-TURRAX stirring speed (13,500–24,000 rpm).

Independent Variables (Factors)			Dependent Variables (Responses)	
CS Conc (% *w*/*v*)	CS-OA Ratio	Stirring Rate (rpm)	Dimensions nm (±SD)	EE% (±SD)
−1	−1	−1	1829 (±303)	70.83 (±1.31)
1	−1	−1	610 (±47)	80.92 (±0.06)
−1	1	−1	627 (±16)	85.42 (±1.72)
1	1	−1	591 (±26)	89.58 (±0.09)
−1	−1	1	1186 (±197)	68.35 (±7.37)
1	−1	1	832 (±93)	79.39 (±2.54)
−1	1	1	634 (±19)	72.19 (±5.60)
1	1	1	620 (±24)	86.51 (±1.33)
0	0	0	760 (±23)	71.28 (±0.80)
0	0	0	759 (±17)	72.68 (±2.04)
0	0	0	733 (±32)	76.62 (±1.36)

**Table 2:** ANOVA table obtained from the statistical analysis of the 2^3^ full factorial design for the effects of the three factors considered and of their binary interactions on NP dimensions.

Source	Sum of Squares	d.f.	Mean Square	F-Ratio	*p*-Value
A: CS concentration	329,266	1	329,266	11.13	0.0290
B: CS:OA ratio	492,528	1	492,528	16.64	0.0151
C: rpm	18,528.1	1	185,28.1	0.63	0.4731
AB	289,941	1	289,941	9.80	0.0352
AC	98,346.1	1	98,346.1	3.32	0.1424
BC	26,106.1	1	26,106.1	0.88	0.4008
Total error	118,385	4	29,596.2		
Total (corr.)	1.3731 × 10^6^	10			

R^2^ = 91.3752 percent; R^2^ (adjusted for d.f.) = 78.4381 percent.

**Table 3:** ANOVA table obtained from the statistical analysis of the 23 full factorial design for the effects of the three factors considered and their binary interactions on encapsulation efficiency (EE%).

Source	Sum of Squares	d.f.	Mean Square	F-Ratio	*p*-Value
A: CSconcentration	195.991	1	195.991	10.24	0.0240
B: CS-OA ratio	146.308	1	146.308	7.64	0.0396
C: stirring	51.6612	1	51.6612	2.70	0.1614
AC	15.4429	1	15.4429	0.81	0.4103
BC	18.8691	1	18.8691	0.99	0.3664
Total error	95.7231	5	19.1446		
Total (corr.)	523.996	10			

R^2^ = 81.7321 percent; R^2^ (adjusted for d.f.) = 63.4641 percent.

**Table 4:** Characterization of CS-OA-stabilized NPs prepared with acetone and comparison with PVA stabilized NPs, unloaded and loaded with curcumin (mean ± SD, *n* = 3).

	Dimensions (nm)	Poly Dispersion Index	Zeta Potential (mV)	EE (%)	Curcumin Concentration (µg/mL)
CS-OA PLGA	346 ± 88	0.32 ± 0.10	59.0 ± 2.34	-	-
PVA PLGA	310 ± 74	0.68 ± 0.15	−26.33 ± 1.52	-	-
Cur-CS-OA PLGA	329 ± 42	0.501 ± 0.016	35.45 ± 3.35	68.75 ± 0.83	82.5 ± 0.99
Cur-PVA PLGA	274 ± 9	0.648 ± 0.083	−0.09 ± 2.4	81.99 ± 1.58	98.1 ± 1.47
